# Genome-Wide Identification and Expression Analysis of m^6^A Regulators in *Bursaphelenchus xylophilus* Across Developmental and Stress Conditions

**DOI:** 10.3390/biology15100786

**Published:** 2026-05-15

**Authors:** Wenhui Guo, Xiaoxiao Xing, Yuke Ma, Bao Li, Huijuan Yin, Jingjing Zhang, Kongshu Ji, Qiong Yu

**Affiliations:** 1 State Key Laboratory of Tree Genetics and Breeding, Nanjing Forestry University, Nanjing 210037, China; gwenhui1106@163.com (W.G.); 18221039447@163.com (X.X.); mayuke2001@163.com (Y.M.); libao131411@163.com (B.L.); yhuijuan2000@163.com (H.Y.); jjzhang@njfu.edu.cn (J.Z.); ksji@njfu.edu.cn (K.J.); 2 Co-Innovation Center for Sustainable Forestry in Southern China, Nanjing Forestry University, Nanjing 210037, China; 3 Beijing National Laboratory for Molecular Sciences, Beijing 100190, China

**Keywords:** *N*^6^-methyladenosine, epitranscriptomics, plant-parasitic nematode, pine wilt disease, stress adaptation

## Abstract

m^6^A represents a prevalent epitranscriptomic modification in eukaryotes. The dynamic balance of m^6^A modification is governed by methyltransferases (writers), demethylases (erasers), and binding proteins (readers). m^6^A regulators are integral to critical biological processes, including embryonic development, cell differentiation, and stress responses. *B. xylophilus*, a highly destructive invasive plant-parasitic nematode, has caused considerable ecological and economic damage worldwide. However, the m^6^A regulatory system in PWNs has not yet been investigated. In this study, we systematically identified 21 m^6^A regulators in PWNs, including 10 writers, 6 erasers, and 5 readers, which belong to the METTL, ALKBH, and KH/RRM families. Phylogenetic and domain analyses revealed the evolutionary conservation and functional diversification of these protein families. Expression profiling indicated stage-specific expression patterns of m^6^A regulators during the egg, larval, diapause, and adult stages. Furthermore, significant responses were observed under low-temperature treatment, β-pinene exposure, and infection of *Pinus thunbergii* seedlings, with *ALKBH* family members exhibiting upregulation under all three stress conditions. Notably, unlike most eukaryotes, the PWN lacks canonical FTO/ALKBH5 demethylases and YTH-domain readers, instead relying on ALKBH6/8 and KH/RRM proteins. These findings suggest that this non-canonical m^6^A regulatory mechanism may contribute to the development and pathogenesis of *B. xylophilus*.

## Simple Summary

The pine wood nematode (PWN), *Bursaphelenchus xylophilus*, causes severe damage to pine forests worldwide, yet the molecular mechanisms controlling its gene activity remain poorly understood. In this study, we investigated *N*^6^-methyladenosine (m^6^A), an RNA common chemical modification that functions as a regulatory switch controlling gene expression. We identified 21 genes in *B. xylophilus* that are involved in adding, removing, or recognizing this modification. These genes showed distinct expression patterns across different developmental stages, from eggs to adults. In addition, they also responded significantly to environmental challenges, including low temperature, exposure to host defense compounds, and infection of pine seedlings. Together, these findings provide fundamental insights into m^6^A–mediated regulation in PWNs and establish a basis for future studies on pine wilt disease.

## 1. Introduction

RNA modifications represent fundamental post-transcriptional regulatory mechanisms, with more than 100 distinct types, including m^6^A and 5-methylcytosine (m^5^C) [1]. Among them, m^6^A, which involves methylation at the N6 position of adenine, is the most prevalent modification in eukaryotic RNAs and has been found across mRNA, lncRNA, circRNA, rRNA, and snRNA [2].

m^6^A is a reversible and dynamic RNA modification regulated by methyltransferases (writers), demethylases (erasers), and binding proteins (readers) [3]. The canonical m^6^A methyltransferases include the core subunits METTL3 and METTL14, along with adaptor proteins WTAP, VIRMA, ZC3H13, HAKAI, and RBM15/15B [3]. Additional writers have been identified, including METTL16 targeting U6 snRNA and *MAT2A* mRNA, METTL5 and ZCCHC4 catalyzing rRNA m^6^A modification, and TMT1A acting on lncRNA. These findings collectively highlight the potential roles of writers in regulating non-coding RNA modification [4,5,6]. The intracellular demethylation reaction is primarily mediated by two demethylase enzymes: FTO and ALKBH5 [7,8]. Both enzymes are eukaryotic homologs of the *Escherichia coli* AlkB DNA demethylase, belonging to the ALKBH family, which require α-ketoglutaric acid (α-KG) and Fe^2+^ as cofactors to catalyze the demethylation of m^6^A modifications in nucleic acids [8]. m^6^A binding proteins include the YTH domain family (YTHDF1/2/3, YTHDC1/2), as well as other direct or indirect binding proteins, including heterogeneous nuclear ribonucleoproteins (HNRNPs), insulin-like growth factor-2 mRNA-binding proteins 1, 2, and 3 (IGF2BP1–3), FMR1, and ELAVL1 [9,10,11]. YTH family proteins bind the RRACH motif in m^6^A-methylated regions via the conserved YTH domain [12]. HNRNPs and ELAVL1 recognize m^6^A modifications through RNA structural remodeling [10,13]. IGF2BP1–3 and FMR1 selectively recognize m^6^A-containing RNAs through their K homology (KH) domains, thereby promoting RNA translation and stability [9,14]. Collectively, m^6^A regulators dynamically modulate various physiological processes through their involvement in methylation modification [15].

Although the m^6^A regulatory machinery has been extensively characterized in mammals and plants, current knowledge in nematodes is predominantly derived from the model organism *Caenorhabditis elegans* (*C. elegans*). In *C. elegans*, m^6^A modification is primarily enriched in non-coding RNAs [16]. *METTL5* (*C38D4.9*) and *ZCCHC4* (*F33A8.4*) are responsible for m^6^A methylation of the small and large ribosomal RNA subunits, respectively. Simultaneous disruption of these two genes dramatically decreases global m^6^A levels, nearly to the limit of detection, and leads to pronounced defects in fertility [16]. Notably, the *zcchc-4* mutant exhibits extended lifespan and widespread transcriptomic dysregulation [16]. *METTL16* (*mett-10*) and *METTL4* (*C18A3.1*) are involved in the methylation of U6 and U2 snRNAs, respectively, and function to suppress germline proliferation [5,17,18]. Further investigations have revealed that METTL5–catalyzed 18S rRNA methylation enhances the translation of *cyp-29A3* mRNA, promoting lipid oxidation and eicosanoid production, which are essential for maintaining normal stress sensitivity in nematodes [19]. *METTL16* (*mett-10*) deposits m^6^A modifications at the 3′ splice site of SAM synthetase genes, directly inhibiting splicing and coordinating nematode metabolic and developmental processes [20]. These studies have established a crucial foundation. Although both PWN and *C. elegans* belong to the class Chromadorea, they belong to different orders, PWN to Aphelenchida (plant-parasitic) and *C. elegans* to Rhabditida (free-living), with an estimated divergence time exceeding 100 million years. Genomic comparisons reveal that core homologous genes are relatively conserved between the two species, while their genomic synteny is poorly maintained [21]. Given these profound evolutionary and ecological differences, whether the m^6^A regulatory system in PWNs is conserved, divergent, or uniquely adapted remains unknown.

Pine wilt disease (PWD), caused by PWNs, induces rapid mortality in pine trees and poses a significant threat to global forest ecosystems [22]. Research efforts have increasingly focused on the pathogenicity and dissemination mechanisms of PWN, particularly the molecular basis underlying its environmental adaptation, host invasion, and colonization. As a migratory endoparasitic nematode, PWN secretes effectors to disrupt host defenses during infection [23]. For instance, BxNMP1 inhibits pine salicylic acid defense pathways by targeting PtTLP-L2 [24], while *BxlTLP1* suppresses ROS scavenging to interfere with immune responses [25]. Following invasion, PWNs feed on xylem parenchyma cells, triggering a robust defense response in pines that results in substantial terpenoid synthesis [26]. Among these, α-pinene and β-pinene are major components of pine defensive terpenoids, which enhance resistance against biotic stressors such as fungi, bacteria, and nematodes [27]. However, studies indicate that high concentrations of α-pinene or β-pinene significantly promote population growth [28,29]. In parallel, PWN invasion involves rapid adaptation to temperature fluctuations, novel hosts, and adverse environmental conditions. Recent multi-omics analyses have revealed that PWN populations invading northern China have evolved a dual strategy for low-temperature adaptation: modulation of cell membrane fluidity via lysophosphatidylethanolamine, and maintenance of genetic material stability through a synergistic chaperone system involving glycosylceramides [30]. Furthermore, the widespread presence of 5-methylcytosine (^5^mC) and N^6^-methyladenine (^6^mA) in the PWN genome suggests that epigenetic modifications may play a role in rapid adaptation to diverse environments and hosts during invasion [31].

In summary, the rapid spread and extensive damage caused by PWNs are closely associated with the regulation of genes involved in detoxification, stress responses, and development. However, current understanding of the molecular mechanisms underlying PWN pathogenicity remains limited, particularly at the level of epitranscriptomic regulation. As a prominent RNA modification in eukaryotes, m^6^A plays critical roles in RNA processing and translation. We hypothesize that m^6^A modification is functionally involved in PWN development and stress adaptation, potentially through lineage–specific regulatory components—but direct evidence is entirely lacking. In this study, we systematically identified m^6^A regulatory genes in the PWN genome and characterized their expression patterns across different developmental stages and stress conditions. This work provides a theoretical foundation for understanding the regulatory role of m^6^A modification in the pathogenicity of PWN, offers insights for developing foundational control strategies against PWD, and establishes a basis for identifying novel molecular targets for disease management.

## 2. Materials and Methods

### 2.1. Nematodes Cultivation and Collection

To characterize the expression patterns of m^6^A regulators under low temperatures, experiments were conducted using the *B. xylophilus* isolates sourced from diseased Yunnan pine logs originating in Zhaotong City, Yunnan Province. The *Botrytis cinerea* strain used in this study was a laboratory-preserved isolate from the Forest Protection Research Laboratory at Nanjing Forestry University and was cultured on PDA (Potato Dextrose Agar) medium at 26 °C ± 1 °C. Once *B. cinerea* had fully colonized the Petri dishes, PWNs were inoculated onto the fungal culture and incubated. Nematodes were collected using the Baermann funnel technique after the mycelium had nearly consumed the agar medium. Mixed-age (L2, L3 and L4) PWNs were washed, resuspended, and then aliquoted into 1.5 mL centrifuge tubes. These tubes were subjected to either a 4 °C incubation for the low-temperature treatment group or a 25 °C incubation for the ambient temperature control group. Samples were collected at 0, 6, 12, and 24 h post-treatment. After incubation, the nematode samples within the centrifuge tubes were centrifuged at 4 °C (pre-cooled) or 25 °C (pre-warmed) to pellet the nematodes. The supernatant was discarded, and the tubes were immediately flash-frozen in liquid nitrogen and stored at −80 °C for subsequent analysis.

### 2.2. Screening and Molecular Characterization of m^6^A Regulators in PWNs

The latest whole-genome data of PWNs (GCA_904066235.2) and the m^6^A regulator protein sequences of *C. elegans* and humans were downloaded from the NCBI database. First, the reported m^6^A regulators sequences of *C. elegans* and humans were used as queries for BLASTp analysis (https://blast.ncbi.nlm.nih.gov/Blast.cgi, accessed on 12 January 2026) against the PWN genome (E-value < 1 × 10^−5^, sequence identity > 30%) [32], yielding a set of candidate sequences. Subsequently, HMM profiles of m^6^A-related conserved domains, including MT-A70 (PF05063), Methyltransf_10 (PF05971), N6-adenine methylase (PF10237), WTAP (PF17098), Virilizer (PF22575), 2OG-Fe (II) oxygenase superfamily (PF13532), YTH (PF04146), and KH domain (PF00013), were obtained from the InterPro database (https://www.ebi.ac.uk/interpro, accessed on 12 January 2026). These profiles were used to search against the PWN protein database using HMMER 3.3.2 (http://hmmer.org/, accessed on 12 January 2026) to identify candidate proteins containing the above conserved domains. The results from both BLAST and HMMER searches were merged, and duplicate sequences were removed using TBtools-II (Toolbox for Biologists) v2.475. The candidate sequences were then submitted to NCBI CDD (CD-Search, https://www.ncbi.nlm.nih.gov/Structure/cdd, accessed on 15 January 2026) for conserved domain validation. Sequences were retained only if they met all of the following criteria: (i) E-value ≤ 1 × 10^−5^; (ii) domain alignment coverage ≥ 50%; and (iii) the conserved functional residues of the target domain (MT-A70, Methyltransf_10, N6-adenine methylase, WTAP, Virilizer, 2OG-Fe (II), YTH, or KH) were intact without deletion of key conserved regions. Through this pipeline, we ultimately identified 21 potential members of the m^6^A regulators in PWNs. Computational characterization of PWN m^6^A regulators was performed using the online tool Expasy (https://www.expasy.org, accessed on 15 January 2026) to determine physicochemical properties, including amino acid length, molecular weight, isoelectric point (pI), instability index, aliphatic index, and grand average of hydropathicity (GRAVY). Subcellular localization was predicted using WOLF PSORT (https://wolfpsort.hgc.jp, accessed on 15 January 2026).

### 2.3. Chromosomal Localization and Protein Structure Analysis

Genomic annotation information for m^6^A regulators in PWNs, including chromosomal location, gene coordinates, and gene length, was obtained from the PWN genome annotation file. Gene density information was extracted using TBtools-II (Toolbox for Biologists) v2.475, and a chromosome localization map of PWN m^6^A genes was subsequently generated with gene density distribution. To further characterize the structural features of PWN m^6^A regulators, secondary structure analysis was performed using SOPMA (https://npsa.lyon.inserm.fr/cgi-bin/npsa_automat.pl?page=/NPSA/npsa_sopma.html, accessed on 2 March 2026). Tertiary structure prediction was carried out using the SWISS-MODEL server (https://swissmodel.expasy.org/interactive, accessed on 2 March 2026). The propensity for liquid–liquid phase separation (LLPS) was predicted using the PLAAC platform (http://plaac.wi.mit.edu/, accessed on 8 March 2026), which identifies prion-like domains (PrLDs) and intrinsically disordered regions (IDRs) associated with phase separation.

### 2.4. Phylogenetic Tree Construction and Gene Structure Analysis

To elucidate the evolutionary relationships between PWNs and other species, multiple sequence alignments of PWN m^6^A family protein sequences with those from different species were performed using MUSCLE in MEGA 11.0. Phylogenetic trees for the three gene categories (writers, erasers, and readers) were subsequently constructed using the neighbor-joining method with 1000 bootstrap replicates. The resulting phylogenetic trees were annotated and visualized using the iTOL website (https://itol.embl.de, accessed on 3 March 2026), and gene nomenclature was assigned based on the clustering results of the phylogenetic trees. Protein domain architectures were analyzed using NCBI CDD (https://www.ncbi.nlm.nih.gov/Structure/cdd/cdd.shtml, accessed on 3 March 2026) with an E-value cutoff < 0.01. Conserved motif distributions of PWN m^6^A proteins were characterized using the MEME suite (https://meme-suite.org/meme, accessed on 3 March 2026). Exon–intron structures of PWN m^6^A genes were extracted from the PWN genome annotation file. Finally, the phylogenetic trees, conserved domains, and gene structures were integrated and visualized using TBtools-II (Toolbox for Biologists) v2.475. The protein sequences of m^6^A regulators from PWNs and other species used for phylogenetic analysis are provided in Table S1.

### 2.5. Total RNA Isolation and m^6^A Regulators Expression Profiling

Total RNA was extracted using M5 SuperPure Total RNA Extraction Reagent (SuperTRIgent, Mei5 Biotechnology, Co., Ltd., Beijing, China). First-strand cDNA was synthesized from 1 μg of total RNA using the Yeasen 1st Strand cDNA Synthesis Kit (Yeasen Biotechnology (Shanghai), Co., Ltd., Shanghai, China), and the resulting cDNA was diluted 1:20 prior to analysis. Quantitative real-time PCR (qRT-PCR) was performed in 10-μL reaction volumes comprising: 10% cDNA template, 4% each of forward and reverse primers (10 μM), 50% SYBR Green Master Mix (Yeasen Biotechnology (Shanghai) Co., Ltd., Shanghai, China), and 32% nuclease-free water. The thermal cycling conditions consisted of an initial denaturation at 95 °C for 2 min, followed by 40 cycles of 95 °C for 10 s and 60 °C for 30 s, with melt curve analysis performed to verify amplification specificity. The experiment was conducted with three technical replicates across three biological replicates. The *β-actin* gene was used as an internal control (Table S2), which has been widely adopted as a stable reference gene in PWNs under low-temperature stress [33], and relative expression levels were calculated using the 2^−ΔΔCT^ method.

### 2.6. RNA-Seq and Statistical Analysis

To investigate the expression patterns of PWN m^6^A regulators across different biological contexts, three publicly available RNA-seq datasets were integrated in this study: different developmental stages (PRJDB3458) [34], after inoculation into *P. thunbergii* seedlings (PRJNA397001) [35], and under varying concentrations of β-pinene stress (PRJNA640733) [29]. For each dataset, raw sequencing reads were processed on the Galaxy platform (https://usegalaxy.com, accessed on 10 March 2026) using the Salmon tool. The Salmon index was constructed based on the PWN CDS sequences, including the annotated CDS sequences of the 21 identified m^6^A regulators. Transcript abundance TPM values were extracted from Salmon quantification outputs, and the TPM values of three biological replicates under each condition were averaged for subsequent analysis. The averaged TPM values were then log_2_-transformed as log_2_ (TPM + 1) and subjected to row-wise Z-score normalization. Expression heatmaps were generated using the built-in Heatmap tool in TBtools-II (Toolbox for Biologists) v2.475.

For each experimental condition, three biological replicates were performed. Data are expressed as mean ± standard error (SE), with error bars in bar graphs representing SE (heatmaps display raw values without error indicators). Analysis of variance (ANOVA) was conducted using SPSS 27.0. The figures were created using GraphPad Prism 10.1.2.

## 3. Results

### 3.1. Identification and Characterization of m^6^A Regulators in PWNs

Following BLASTX

(https://blast.ncbi.nlm.nih.gov/Blast.cgi?PROGRAM=blastx, accessed on 12 January 2026) search and conserved domain verification, 21 m^6^A regulators were identified within the PWN genome. These genes were systematically named based on their phylogenetic relationships with *C. elegans* homologs, resulting in the classification of 10 writers, 6 erasers, and 5 readers (Table 1). Physicochemical property analysis revealed considerable variation in amino acid length, molecular weight, and isoelectric point among these proteins.

The lengths of writer proteins ranged from 219 aa (BxTMT1A) to 2919 aa (BxRBM15b), with corresponding molecular weights ranging from 24.97 to 321.18 kDa and isoelectric points varying from 4.88 to 9.35, implying diverse subcellular milieus and electrostatic interaction partners. Notably, BxRBM15b is exceptionally large, suggesting that this protein may serve as a multi-domain scaffold, whereas BxTMT1A and BxMETTL5a are relatively small, likely representing core catalytic units. For erasers, the proteins ranged from 259 aa (BxALKBH6a) to 941 aa (BxALKBH6b), with molecular weights ranging from 29.03 to 107.54 kDa and isoelectric points varying from 5.40 to 8.94. Reader proteins ranged from 364 aa (BxHNRNP) to 522 aa (BxKHSRP), with molecular weights ranging from 40.00 to 55.67 kDa and isoelectric points ranging from 6.03 to 9.17. Stability analysis indicated that BxMETTL5a, BxMETTL14b, BxMETTL16, BxZCCHC4, BxTMT1A, BxALKBH8c, BxALKBH8d, and BxFMR1 are stable proteins, while the remaining proteins are unstable. Unstable proteins may be subject to rapid turnover or post-translational regulation, potentially enabling dynamic control of m^6^A modification in response to environmental cues. All proteins exhibited negative GRAVY values, indicating their hydrophilic nature. RNA-binding proteins require this property to interact with the solvent-accessible RNA backbone in aqueous environments. Subcellular localization predictions suggested that writers are primarily distributed in the nucleus, cytoplasm, and plasma membrane, consistent with their proposed roles in co-transcriptional m^6^A deposition and RNA processing; most erasers localize to the nucleus, indicating that demethylation may primarily occur in the nucleus prior to mRNA export; and among readers, BxELAVL1a and BxELAVL1b localize to the nucleus, with others localizing to the cytoplasm, suggesting potential functions in post-transcriptional regulation, such as mRNA stability control and translation efficiency modulation.

### 3.2. Chromosomal Localization of m^6^A Regulators in PWNs

**Figure 1:**
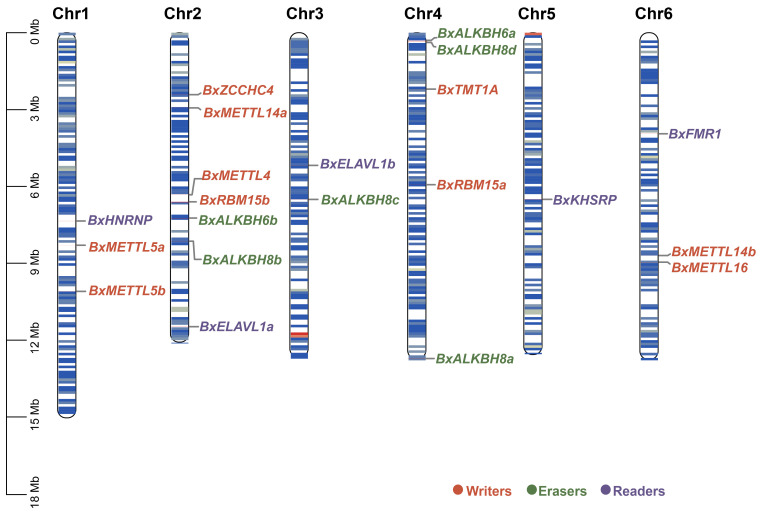
Chromosomal locations of the identified m^6^A regulators in PWNs, color-coded by functional category: writers in orange, readers in green, and erasers in purple. The scale bar on the left indicates chromosome lengths in megabases (Mb). Chromosome numbers are shown above each chromosome.

The 21 m^6^A regulators were unevenly distributed across the six chromosomes of PWNs (Figure 1). Chromosome 2 harbored the highest gene density, containing 4 writers (*BxZCCHC4*, *BxMETTL14a*, *BxMETTL4*, *BxRBM15b*), 2 erasers (*BxALKBH6b*, *BxALKBH8b*), and 1 reader (*BxELAVL1a*). Chromosome 4 followed with 2 writers (*BxTMT1A*, *BxRBM15a*) and 3 erasers (*BxALKBH6a*, *BxALKBH8a*, *BxALKBH8d*). Chromosomes 1 and 6 each contained 3 genes, Chromosome 3 contained 2 genes (*BxELAVL1b*, *BxALKBH8c*), and Chromosome 5 contained 1 gene (*BxKHSRP*). Notably, erasers were present on Chromosomes 2, 3, and 4, with two genes on Chromosome 4 arranged in tandem, suggesting a tandem duplication events during evolution. In contrast, writers were widely distributed across Chromosomes 1, 2, 4, and 6, exhibiting a dispersed pattern that reflects the evolutionary complexity of the methyltransferases.

### 3.3. Phylogenetic Analyses of m^6^A Writers, Readers and Erasers

**Figure 2:**
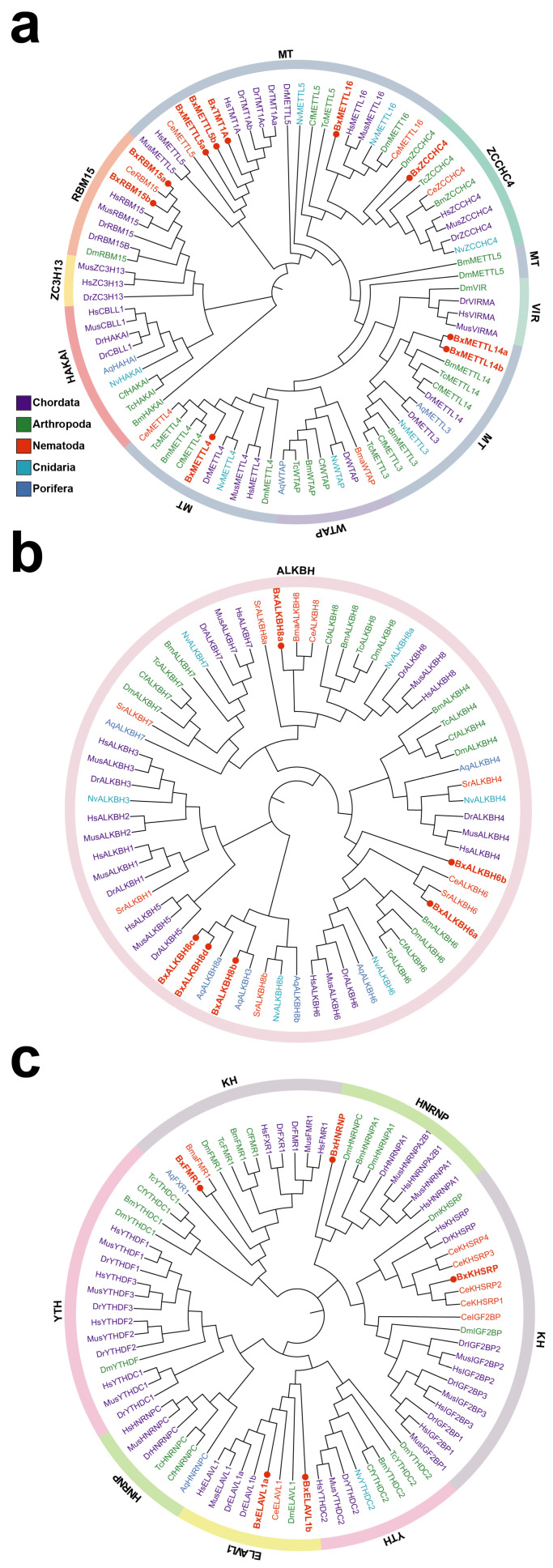
Phylogenetic analysis of m^6^A regulators in PWNs and other species. The neighbor-joining (NJ) trees depict the evolutionary relationships of m^6^A writers (**a**), erasers (**b**), and readers (**c**), with different families clades shown in distinct colors. Different phyla are color-coded: Chordata (purple), Arthropoda (green), Nematoda (red), Cnidaria (cyan), and Porifera (blue). Trees were generated with 1000 bootstrap iterations, and all PWN regulators are indicated by red circles. The first two letters of gene names in the tree refer to Latin names of various species. Bx: *Bursaphelenchus xylophilus*; Ce: *Caenorhabditis elegans*; Sr: *Strongyloides ratti*; Bma: *Brugia malayi*; Nv: *Nematostella vectensis*; Aq: *Amphimedon queenslandica*; Cf: *Camponotus floridanus*; Bm: *Bombyx mori*; Tc: *Tribolium castaneum*; Dm: *Drosophila melanogaster*; Dr: *Danio rerio*; Mm: *Mus musculus*; Hs: *Homo sapiens*.

To investigate the molecular evolution of m^6^A regulators, phylogenetic analysis was performed on PWNs and other species. Based on protein sequences, neighbor-joining (NJ) methods were employed to construct phylogenetic trees for m^6^A writers, erasers, and readers, respectively (Figure 2). The analysis encompassed representative species from Chordata, Arthropoda, Nematoda, Cnidaria, and Porifera. The m^6^A writer tree was divided into four clades, corresponding to six gene families: MT, ZCCHC4, WTAP, VIR, HAKAI, ZCCHC3 and RBM15 (Figure 2a). The MT group further included the METTL3, METTL4, METTL5, METTL14, METTL16, and TMT1A (METTL7A) subgroups. Evolutionary conservation and lineage specificity. BxMETTL4, BxMETTL14a/b, and BxZCCHC4 clustered with arthropod orthologs, suggesting that these genes have been evolutionarily conserved within the Ecdysozoa clade (which includes both arthropods and nematodes). In contrast, BxMETTL16 and BxTMT1A clustered with vertebrate orthologs. BxMETTL5a/b and BxRBM15a/b formed distinct nematode-specific branches with *C. elegans* orthologs, implying lineage-specific expansion or functional divergence of these genes within the Nematoda phylum. All m^6^A erasers belonged to the *ALKBH* gene family and were divided into four groups (Figure 2b). Within the ALKBH8 branch, orthologous sequences from PWN, *Strongyloides ratti*, *Actinia stricta*, and *Leucosolenia complicate* were densely distributed in the tree, forming an independent subcluster that closely aligned with the taxonomic relationships of these species. This phylogenetic pattern suggests that the ALKBH8 subfamily has undergone ancient diversification, with PWN retaining ancestral copies that have subsequently diverged from their counterparts in model organisms. The readers were classified into three branches, corresponding to the YTH domain family (YTHDC1/2, YTHDF1/2/3), the KH domain family (IGF2BP, KHSRP, FMR1), and the HNRNP family (HNRNPC, HNRNPA/B), along with the ELAVL1 family (Figure 2c). The first branch comprised the YTH family, ELAVL1, and HNRNPC, where BxELAVL1a/b clustered with nematode and arthropod orthologs. The second branch included IGF2BP, KHSRP, and HNRNPA/B, with BxKHSRP forming a nematode-specific branch with *C. elegans* orthologs and BxHNRNP clustering with arthropod orthologs. The third branch consisted of FMR1 and YTHDC1, with BxFMR1 clustering with *Brugia malayi* orthologs, raising the possibility that FMR1-mediated m^6^A recognition is associated with parasitic lifestyles. Overall, the phylogenetic trees show that some m^6^A regulators (e.g., METTL16 and TMT1A) are evolutionarily conserved across animals, whereas others (e.g., METTL5, RBM15, and KH domain readers) have undergone nematode–specific expansion, and YTH domain readers are absent in PWNs.

### 3.4. Conserved Motifs and Gene Structure Analysis of m^6^A Regulators

**Figure 3:**
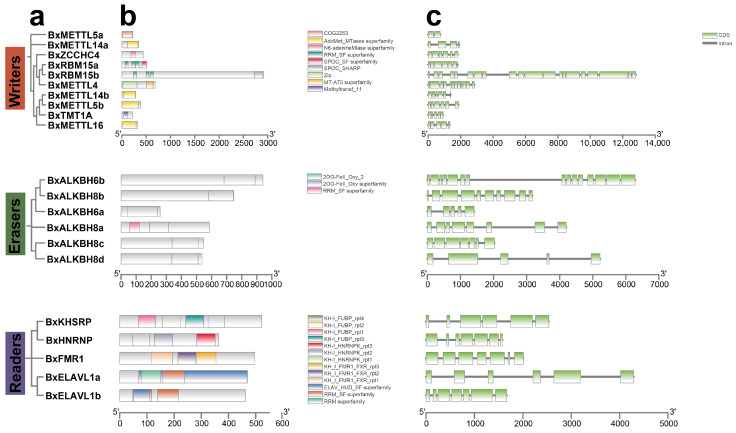
Phylogenetic tree, domain architecture, and gene structure of m^6^A regulators in PWNs. (**a**) Phylogenetic tree of m^6^A regulators; (**b**) Functional protein domains; (**c**) Gene structure.

**Figure 4:**
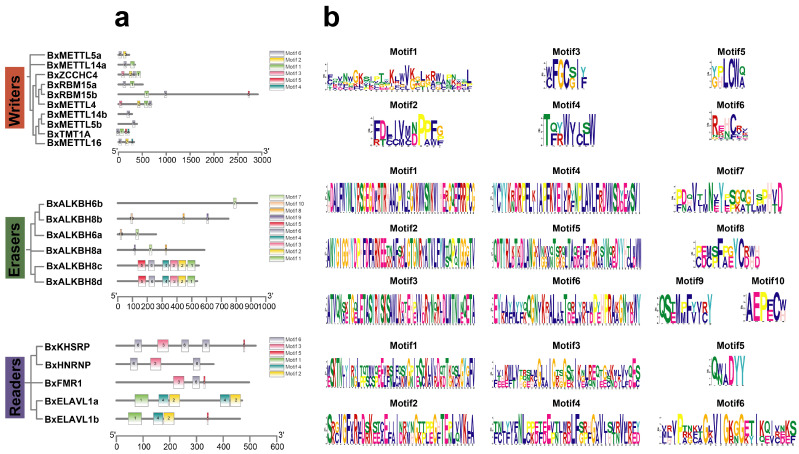
Conserved motifs and sequence logos of m^6^A regulators in PWNs. (**a**) Conserved sequence motifs; (**b**) Sequence logos showing the conserved amino acid residues at each motif position. The height of each letter is proportional to the frequency of the corresponding amino acid residue at that position.

To elucidate the sequence–function relationships of m^6^A regulators in PWNs, a systematic analysis was conducted on phylogenetic tree (Figure 3a), conserved domain architecture (Figure 3b), gene structure (Figure 3c), and motif organization (Figure 4a,b).

Domain analysis revealed the characteristic structural features of these writers (Figure 3b). Core methyltransferases (BxMETTL16, BxMETTL14a/b, BxMETTL5b) all harbored the AdoMet_MTases superfamily domain (CDD: cl17173), which provides the SAM-binding site essential for methyl group transfer. Additionally, BxMETTL5a possessed the COG2263 domain (CDD: COG2263), a family predicted to function as an RNA methyltransferase. BxMETTL4 contained both the Zip domain (CDD: pfam02535) and the MT-A70 superfamily domain (CDD: pfam05063), a combination that may facilitate protein–protein interactions within the methyltransferase complex. The auxiliary regulatory factors BxRBM15a/b exhibited lineage-specific domain combinations, including RRM_SF (CDD: cl17169) and SPOC_SF (CDD: cl45902). BxTMT1A harbored the Methyltransf_11 domain (CDD: pfam08241), and BxZCCHC4 contained the N6-adenineMlase superfamily domain (CDD: pfam10237). In erasers, ALKBH family universally conserved the 2OG-Fe (II) oxygenase domain (PF13532), the core module for their catalytic m^6^A demethylation activity. Regarding readers, m^6^A reader genes were classified into two subfamilies: the KH domain family (BxKHSRP, BxHNRNP, BxFMR1) and the ELAVL1 family (BxELAVL1a, BxELAVL1b). KH domains are known to selectively recognize m^6^A modified RNAs to regulate mRNA stability and translation.

Gene structure analysis revealed considerable variation among the three regulator types (Figure 3c). The exon numbers of PWN m^6^A writers, erasers, and readers ranged from 2 to 20, 5 to 15, and 6 to 7, respectively. Among the writers, the gene structure was the most diverse. BxRBM15b possessed the most complex structure, containing 20 exons, suggesting potential alternative splicing to generate functional diversity, while BxMETTL5a was the smallest gene, containing only 2 exons. Analysis of conserved motifs in PWN m^6^A writers identified six motifs (motifs 1–6) (Figure 4a). While BxMETTL4, BxZCCHC4, and BxTMT1A possess relatively complete motif sets, other genes exhibit reduced motif compositions. BxALKBH6a contained motif 7 and motif 10, while BxALKBH6b contained only motif 7. The ALKBH8 subfamily displayed the most diverse motif composition. BxALKBH8c/d retained motif 1–6, whereas BxALKBH8a/b contained only motifs 7–10, highlighting clear motif divergence between these subgroups (Figure 4b). The KH domain subfamily shared motif 3 and motif 6, while the ELAVL1 subfamily shared motif 1, motif 2, and motif 4. Their motif arrangements were highly conserved, reflecting evolutionary conservation within the subfamilies.

### 3.5. Protein Structure Prediction

**Figure 5:**
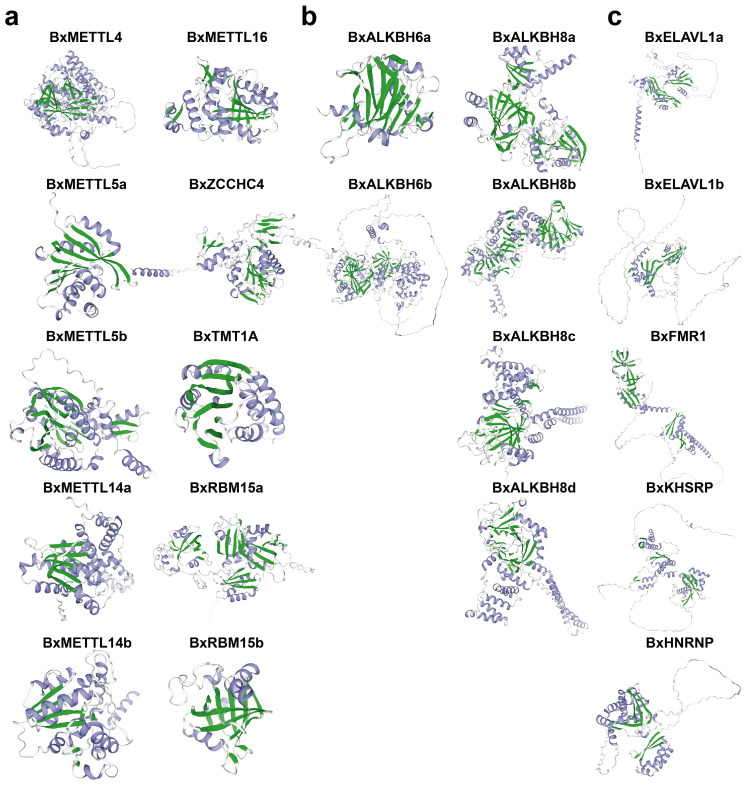
Tertiary structure prediction of m^6^A regulators in PWNs, showing m^6^A writers (**a**), erasers (**b**), and readers (**c**). Color coding: Alpha helices are shown in blue, beta strands in green, and random coils in white.

Secondary structure analysis of m^6^A regulators in PWNs revealed significant differences in composition among different family members (Table S3). Among writers, the proportion of α-helix ranged from 23.64% to 52.51%, with the highest proportion observed in BxTMT1A (52.51%); random coil accounted for 29.68% to 64.06%, with BxRBM15b exhibiting the highest proportion (64.06%). For erasers, α-helix ranged from 29.76% to 56.02%, with BxALKBH8b and BxALKBH8c exceeding 55%; random coil accounted for 32.59% to 51.74%, with both BxALKBH6a and BxALKBH6b exceeding 50%. Readers were predominantly composed of random coil (52.72–63.07%), with relatively lower α-helix content (20.88–30.58%). Tertiary structure prediction revealed distinct spatial conformations among the m^6^A regulators (Figure 5), which corresponded to their RNA binding modes and catalytic mechanisms, suggesting that they perform different biological functions in the regulation of m^6^A modification.

### 3.6. LLPS Propensity of m^6^A Regulators

**Figure 6:**
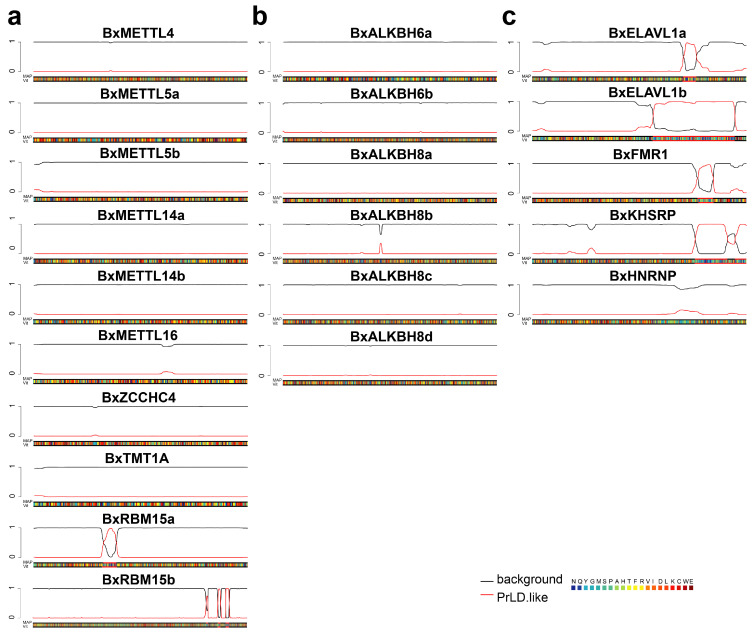
PLAAC analysis reveals potential phase-separating PrLDs and disordered sequences in m^6^A regulators. (**a**) PrLD predictions for m^6^A writers. (**b**) PrLD predictions for m^6^A erasers. (**c**) PrLD predictions for m^6^A readers. Baseline (black) contrasts with predicted PrLD domains (red). Red lines above the baseline suggest the presence of PrLDs, which are likely to undergo phase separation.

Accumulating evidence has demonstrated that m^6^A modification promotes phase separation by enhancing multivalent interactions between RNA and binding proteins, thereby partitioning modified mRNAs into specific membrane-less organelles [36]. Using the PLAAC platform, we predicted prion-like domains (PrLDs) and intrinsically disordered regions (IDRs) in PWN m^6^A regulators (Figure 6). In the PLAAC plots, regions where the red line rises above the black baseline are predicted to contain PrLDs/IDRs, which are known to promote LLPS. Among the 10 writers, only BxRBM15a and BxRBM15b exhibited clear PrLD signals (Figure 6a); none of the six erasers showed significant signals (Figure 6b); and among the five readers, BxELAVL1a, BxELAVL1b, BxFMR1, and BxKHSRP displayed prominent PrLD signals, with BxELAVL1b showing particularly strong and extended regions (Figure 6c). These findings suggest that m^6^A mediated phase separation may coordinate RNA condensate formation in PWNs during environmental stress, thereby enabling rapid gene expression regulation for stress adaptation.

### 3.7. Developmental Expression Pattern of m^6^A Regulators

**Figure 7:**
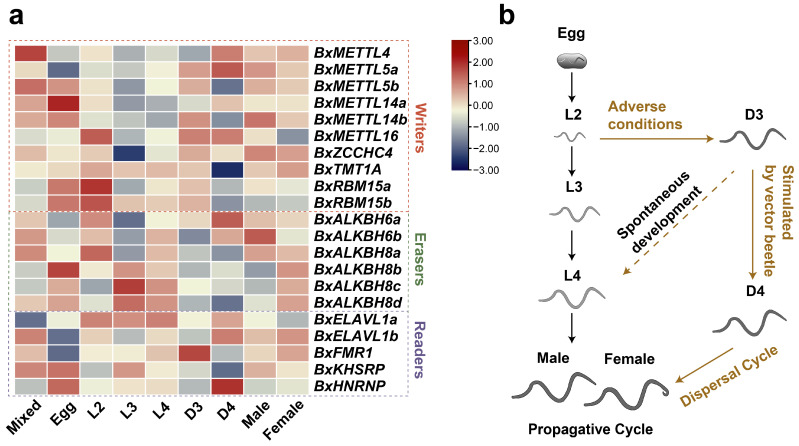
Expression analysis of m^6^A regulators by RNA-seq. (**a**) Expression profiles of m^6^A regulators in different stages, including mixed propagative stages, egg, L2, L3, L4, D3, D4, male, and female. The rows of PWN m^6^A regulators have been clustered and normalized using the Z-score method. The color scale indicates expression levels, with blue representing lower expression and red representing higher expression. (**b**) Life cycle of PWNs, divided into propagative and dispersal cycles. Egg (embryo), L2 (2nd stage larva), L3 (3rd stage larva), L4 (4th stage larva), D3 (3rd stage dispersal juvenile), D4 (4th stage dispersal juvenile), Male, and Female (adult).

To investigate the potential functions of m^6^A regulators in PWN growth and development, transcriptomic expression profiles were analyzed across nine sample stages (Figure 7a,b). Writers (*BxMETTL5b*, *BxMETTL14a/b*, *BxMETTL16*, *BxRBM15a/b*) showed relatively high expression in the egg and L2 stages, which correspond to embryonic development and early larval growth; this suggests that these writers may be involved in initiating m^6^A modifications required for early developmental programs. In contrast, *ZCCHC4* and *TMT1A* were lowly expressed at the L3 and D4 stages, respectively, implying that their functions may be more critical at other phases. Erasers displayed developmentally stage-specific expression patterns. *BxALKBH8a* was specifically highly expressed in the L2 stage, a period of rapid somatic growth and molting preparation, hinting at a role in resetting m^6^A marks during larval transitions. *BxALKBH8b/c/d* exhibited higher expression in the L3 and L4 stages, when gonad development and reproductive system maturation occur, suggesting that these erasers may participate in regulating genes essential for sexual maturation. In contrast, *BxALKBH6a* expression was significantly upregulated in the D4 stage, whereas *BxALKBH6b* reached peak expression in the male stage, implicating a potential role in spermatogenesis or male-specific gene expression. For readers, expression peaks were observed in certain genes during the juvenile stages (D3/D4), with *BxFMR1* highly expressed in the D3 stage and *BxHNRNP* highly expressed in the D4 stage. During these diapause stages, the elevated expression of readers may promote selective translation of mRNAs essential for long-term survival. In contrast, *BxELAVL1a/b* showed significantly low expression in the egg stage, and *BxKHSRP* also exhibited low expression in the D4 stage. The developmental expression heatmap revealed expression divergence among several paralogous gene pairs. For instance, *BxMETTL5a* exhibited low expression in the egg stage, whereas *BxMETTL5b* showed high expression in the same stage. *BxALKBH8a* exhibited high expression in the L2 stage, whereas its gene pairs (*BxALKBH8b/c/d*) showed lower expression in the corresponding stages. These results indicate that m^6^A regulators exhibit dynamic and stage–specific expression patterns, suggesting potential roles in developmental transitions of PWNs.

### 3.8. Expression Patterns of m^6^A Regulators During Host Infection and β-Pinene Stress

**Figure 8:**
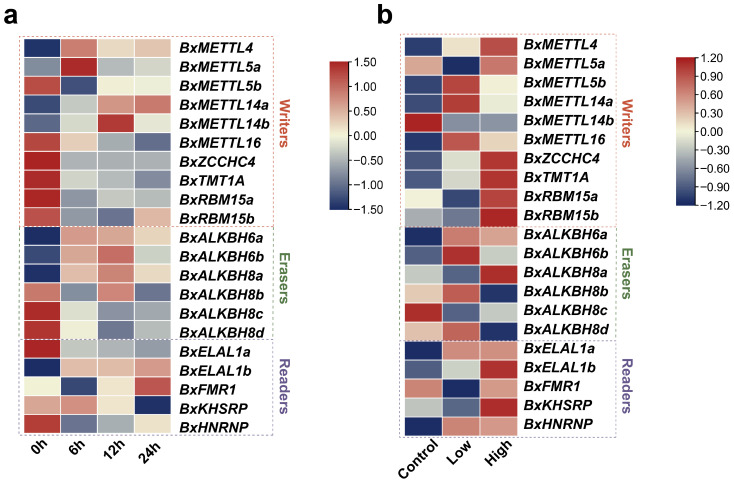
Expression dynamics of m^6^A regulators (**a**) after inoculation into *P. thunbergii* seedlings and (**b**) under β-pinene stress. Low: low-concentration treatment; High: high-concentration treatment. The rows of PWN m^6^A regulators have been clustered and normalized using the Z-score method. The color scale indicates expression levels, with blue representing lower expression and red representing higher expression.

To elucidate the expression patterns of m^6^A regulators in PWNs during host infection and in response to pine-derived metabolites, transcriptomic data following inoculation into *P. thunbergii* and under β-pinene stress were analyzed (Figure 8a,b). During infection, several genes displayed dynamic transcriptional responses (Figure 8a). Prior to inoculation, *BxMETTL5b*, *BxMETTL16*, *BxZCCHC4*, *BxTMT1A*, *BxRBM15a/b*, *BxALKBH8b/c/d*, *BxELAVL1a*, and *BxHNRNP* exhibited high expression levels prior to infection and were significantly downregulated following infection. At the early stage of infection (6 h), *BxMETTL4*, *BxMETTL5a*, *BxALKBH6a/b*, *BxALKBH8a* and *BxELAVL1b* were significantly upregulated. *BxMETTL14a/b* and *BxALKBH8b* were significantly upregulated at the mid-stage of infection (12 h) and maintained relatively high expression levels. At the late stage of infection (24 h), *BxFMR1* exhibited increased high expression.

Under β-pinene stress, most m^6^A regulators exhibited significant differential expression, with response patterns displaying concentration dependence (Figure 8b). Under low-concentration treatment, nine genes (*BxMETTL5b*, *BxMETTL14a*, *BxMETTL16*, *BxALKBH6a/b*, *BxALKBH8b*, *BxALKBH8d*, *BxELAVL1a*, *BXHNRNP*) were significantly upregulated, while five genes (*BxMETTL5a*, *BxRBM15a*, *BxALKBH8a/c*, *BxFMR1*) were significantly downregulated. Under high-concentration treatment, most genes were significantly upregulated, except for three genes (*BxMETTL14b*, *BxALKBH8b*, *BxALKBH8d*) significantly downregulated. Notably, several regulated genes exhibited concentration-specific response patterns. *BxALKBH6a*, *BxELAVL1a*, and *BxHNRNP* showed no significant differences between concentrations, whereas *BxMETTL5b*, *BxMETTL14a*, *BxMETTL16*, *BxALKBH6b*, *BxALKBH8b* and *BxALKBH8d* were upregulated under low-concentration treatment but downregulated under high-concentration treatment. The rest genes showed continuous upregulation tendency. These findings suggest that m^6^A regulators may be involved in the response of PWNs to pine–derived metabolite stress.

### 3.9. Expression Analysis of m^6^A Regulators Under Cold Stress

**Figure 9:**
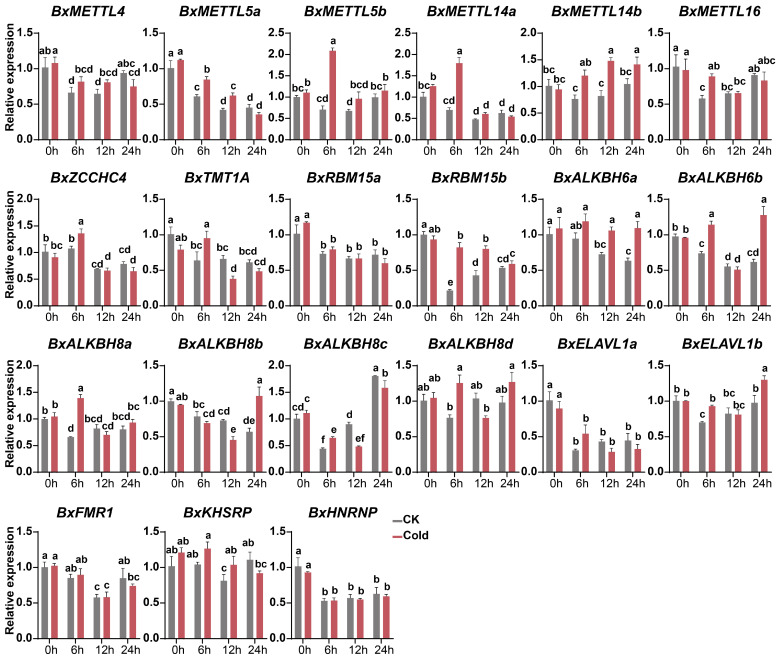
qRT-PCR analysis of relative expression levels of m^6^A regulators in PWNs under 4 °C cold stress. Expression levels of the same gene across different time treatments, including control (CK) groups and cold-treated groups (0, 6, 12, 24 h). The mean (± SE) expression values were calculated from three independent biological replicates and three technical replicates. Different lowercase letters indicate significant differences (*p* < 0.05, one-way ANOVA with LSD and Duncan’s post hoc tests), while the same letters denote no significant difference.

The continued expansion of PWNs into the cold–temperate regions of northern China, where the annual average temperature is below 10 °C, suggests that this species has evolved remarkable low-temperature tolerance. To further evaluate the potential involvement of m^6^A regulators in temperature stress adaptation, RT-qPCR was performed to examine their temporal expression patterns under low-temperature treatment (Figure 9). Overall, most m^6^A regulators exhibited rapid transcriptional responses under cold stress, with the strongest induction observed at 6 h post-treatment. Among them, *BxMETTL5b*, *BxMETTL14a*, *BxZCCHC4*, and *BxALKBH8a* showed significant upregulation and reached peak expression levels at 6 h, whereas *BxMETTL14b* and *BxELAVL1b* exhibited delayed responses and reached their highest expression levels at 24 h. Among the writer genes, most were significantly upregulated at 6 h post-treatment. Specifically, *BxMETTL5b*, *BxMETTL14a*, *BxMETTL16*, *BxZCCHC4*, *BxTMT1A* and *BxRBM15b* exhibited significant upregulation (*p* < 0.05) and reached their peak expression levels at this time point. The rapid and coordinated upregulation of multiple writers at the early stage of cold exposure suggests that m^6^A deposition is quickly activated upon temperature drop, potentially to rapidly modify stress–responsive transcripts, thereby facilitating their processing or stability. At 12 h post-treatment, *BxMETTL5a*, *BxMETTL14b* and *BxRBM15b* remained significantly upregulated compared with the corresponding controls, whereas *BxTMT1A* displayed significant downregulation. The expression levels of most writers decreased over time after peaking at 6 h. By 24 h, only *BxMETTL14b* maintained a relatively high expression level, suggesting that certain writers may sustain m^6^A modification on specific long-lived transcripts required for prolonged cold adaptation. Erasers displayed distinct temporal dynamics. At 6 h post-treatment, *BxALKBH6b*, *BxALKBH8a*, *BxALKBH8c* and *BxALKBH8d* were significantly upregulated. At 12 h post-treatment, the expression of these genes showed clear divergence: *BxALKBH6a* was significantly upregulated; whereas *BxALKBH8b* and *BxALKBH8c* were significantly downregulated, reaching their lowest expression levels across the entire time course. The early upregulation of both writers and erasers indicates a dynamic balance between methylation and demethylation during cold stress, rather than a unidirectional change. This oscillation may allow rapid resetting of m^6^A marks on key transcripts, enabling flexible gene expression reprogramming in response to fluctuating temperatures. In contrast, readers showed relatively stable expression patterns throughout the treatment period, with limited transcriptional variation. Only *BxELAVL1b* was significantly upregulated at 6 h and 24 h. Collectively, m^6^A regulatory genes in PWNs displayed temporal expression patterns under low-temperature stress, indicating that m^6^A modification may play a stage–specific regulatory role in low-temperature adaptation.

## 4. Discussion

### 4.1. Evolutionary Conservation and Non-Canonical Features of m^6^A Regulators in PWNs

In this study, 21 candidate m^6^A regulators were identified in the PWN genome, including m^6^A writers, erasers, and readers, along with their associated family genes. Phylogenetic analysis provided a framework for the systematic nomenclature of these genes and established a foundation for subsequent functional studies. The identified genes were classified into the METTL (writers), ALKBH (erasers), and KH/RRM (readers) families, each comprising multiple members, suggesting functional diversification within these protein families. Notably, several distinctive characteristics were observed, including the absence of canonical m^6^A demethylases and YTH domain-containing readers, as well as the potential involvement of RNA phase separation in post-transcriptional regulation.

Similar to *C. elegans*, typical m^6^A demethylases (FTO/ALKBH5 homologs) were identified in PWNs. Although the *ALKBH* gene family comprises nine members (*ALKBH1*-*ALKBH8* and *FTO*) in mammals, its distribution in invertebrates exhibits significant contraction and lineage–specific diversification [37]. As shown in the phylogenetic tree (Figure 2b), arthropods, including crustaceans and insects, generally lack *FTO* and *ALKBH5* homologs. However, studies have indicated that the distribution of *ALKBH5* varies across insect orders: it is absent in Diptera and Lepidoptera but retained in Coleoptera and Hemiptera [38]. These findings suggest that the absence of canonical m^6^A demethylases is not a unique derived feature of nematodes but rather an ancestral trait widely present in the Ecdysozoa, which includes arthropods and nematodes. This evolutionary context provides critical insights into the selective retention of the ALKBH6 and ALKBH8 subfamilies in PWNs. Studies have confirmed that ALKBH8 exhibits m^6^A demethylase activity in *Aedes aegypti* and *Drosophila melanogaster* [39], while in shrimp, both ALKBH1 and ALKBH8 possess m^6^A demethylation activity, with knockdown of either gene leading to increased global m^6^A levels and overexpression resulting in decreased global m^6^A levels [40]. These findings suggest that ALKBH family members in PWNs may perform alternative demethylation functions in the absence of canonical erasers.

At the recognition level, PWNs also lacks classical YTH domain-containing reader proteins that typically mediate m^6^A recognition in eukaryotes. Instead, numerous proteins containing KH domain and RRM domain were identified in PWNs, suggesting the existence of non-canonical m^6^A recognition mechanisms. In mammals, IGF2BPs selectively bind m^6^A modified RNAs through their KH domains (particularly KH3–4), thereby regulating the stability and translation efficiency of target mRNAs [41]. Additionally, m^6^A influences the local structure of RNA, exposing previously occluded protein-binding motifs and facilitating the binding of RRM domain-containing proteins such as HNRNPs. These structural changes enable RNA-binding proteins to participate in m^6^A–dependent regulatory pathways without directly recognizing the modification itself [42]. Therefore, it is possible that KH/RRM domain-containing proteins in PWNs act as alternative m^6^A readers, forming a non-canonical recognition system that regulates RNA metabolism. Future studies should focus on elucidating whether these KH/RRM proteins recognize m^6^A modified RNAs and clarifying the molecular mechanisms, thereby revealing alternative regulatory pathways for m^6^A recognition in PWNs.

### 4.2. Stage-Specific Expression of m^6^A Regulators During PWN Development

Expression profiling revealed that m^6^A regulators in PWNs exhibit dynamic and developmentally stage-specific expression patterns (Figure 7). Writers are preferentially expressed during early developmental stages (egg to L2), coinciding with embryogenesis and rapid somatic growth. In *C. elegans*, loss of *METTL5* results in reduced fertility [16]. In *D. melanogaster*, methyltransferase complex components including METTL3 (IME4), METTL14, RBM15 (Nito), and Hakai coordinately regulate alternative splicing of the sex determination key gene *Sxl*, and their loss impairs germ cell differentiation and causes ovarian developmental delay [43,44]. Likewise, in mammals, conditional knockout of *METTL3* and *METTL14* in oocytes and spermatogonial stem cells similarly leads to gametogenesis arrest and fertility loss [45,46]. Given their high expression during early developmental stages and the conserved functions of their homologous genes in other species, these writers likely play a critical role in the embryonic and early larval development of PWNs.

Such stage-specific expression of *ALKBH* family members has also been reported in other arthropods. In *Litopenaeus vannamei*, *ALKBH1*, *ALKBH2*, and *ALKBH4* exhibited highest expression during the intermolt stage, whereas *ALKBH6* and *ALKBH8* peaked in the post-molt stage, suggesting their potential roles in regulating molting and ammonia toxicity resistance [40]. These findings suggest that different *ALKBH* members may have specialized demethylation functions during development in PWNs, with *ALKBH8* potentially playing an important role in larval stages and *ALKBH6* involved in diapause and reproductive regulation.

Readers also displayed stage-specific expression, particularly during the diapause stages (D3/D4), implying that m^6^A-mediated RNA regulation may contribute to developmental transitions and metabolic adjustments during dormancy. In *D. melanogaster*, the nuclear reader YT521-B and the cytoplasmic reader CG6422 serve as major m^6^A binding proteins, participating in alternative splicing and cytoplasmic RNA metabolism, respectively [44]. In addition, the cytoplasmic *FMR1* has been identified as an m^6^A reader, capable of specifically recognizing and binding m^6^A modified mRNAs [9]. Although PWN lacks YTH domain-containing readers, given that BxELAVL1a/b are predicted to localize to the nucleus, while BxKHSRP, BxHNRNP, and BxFMR1 are predicted to localize to the cytoplasm (Table 1), it is speculated that similar functional partitioning between nuclear and cytoplasmic compartments may exist. Given that FMR1 is involved in embryonic development in *D. melanogaster* [9], the high expression of *BxFMR1* during the diapause stage in PWNs suggests a potential role in developmental transition, possibly mediating stage-specific translational regulation. The m^6^A modification system in PWNs, through the coordinated action of writers, erasers, and readers, may play an important role in larval development, diapause transition, and reproduction.

### 4.3. Dynamic Responses of m^6^A Regulators to Environmental Stresses

Environmental stress represents another critical challenge for PWN during host colonization and geographic expansion. Survival under stress conditions requires coordinated regulation at both transcriptional and translational levels to maintain critical survival pathways [47]. m^6^A plays a key role in post-transcriptional regulation under stress conditions by modulating RNA processing and translation [48]. During infection of *P. thunbergii*, m^6^A regulators exhibited temporally dynamic expression patterns, with different genes being activated or suppressed at early, middle, and late stages of infection, indicating that these genes participate in a coordinated regulatory network mediating host adaptation. This dynamic pattern is consistent with the regulatory characteristics of m^6^A modification systems during infection of plant-parasitic nematodes [49,50], suggesting that m^6^A modification may be involved in the interaction between PWN and its host through temporal regulation. In addition to host infection, pine-derived chemical defenses, such as β-pinene, also represent a major challenge for PWN. Under β-pinene stress, m^6^A regulators exhibited both concentration–dependent and concentration–independent expression patterns. Previous studies have shown that β-pinene exerts a concentration–dependent effect on PWN, with low concentrations inhibiting reproduction, whereas high concentrations promote reproduction [29]. Genes that exhibited significant changes under low-concentration stress may play important roles in the early stages of nematode perception and response to host defenses, mediating basal detoxification and physiological homeostasis (Figure 8). In contrast, genes induced at higher concentrations may be involved in adaptive reprogramming and reproductive transformation, consistent with the observation that high concentrations of terpenes promote population expansion [28]. Furthermore, *BxAKLBH6*, *BxELAVL1a* and *BxHNRNP*, which showed concentration-independent upregulation, may serve as general stress response factors involved in basal defense, similar to the function of effector proteins (BxVAP1) in the interaction between PWN and its host [51]. These transcriptional responses indicate that m^6^A modification may contribute to the regulation of detoxification metabolism and stress tolerance during host–parasite interactions.

PWN has expanded northward into regions with annual average temperatures below 10 °C, indicating that it has evolved strong low-temperature tolerance [33]. Therefore, elucidating its low-temperature adaptation mechanisms is crucial for predicting invasion risk and developing control strategies. Under cold stress, most m^6^A writers were rapidly upregulated during the early stage of treatment. This early response is consistent with the upregulation of *METTL3* and *METTL14* in *Larimichthys crocea* under low-temperature starvation stress [52], suggesting that the m^6^A system is rapidly activated at the initial stage of cold stress. At later stages, several *ALKBH* family members exhibited stronger transcriptional activation, implying that demethylation processes may become more important during prolonged cold exposure. This temporal expression pattern differs from that of the freeze-tolerant wood frog, in which m^6^A methyltransferase complexes are induced under cold stress while eraser and reader levels are suppressed [53]. Overall, most m^6^A regulators exhibited significant expression changes under various treatments, confirming their important functions in host infection and their capacity to respond to pine-derived metabolites, although the underlying mechanisms remain to be elucidated. Notably, *ALKBH* family members were consistently upregulated under all three stress conditions, further highlighting their potential roles in stress adaptation.

### 4.4. Predicted LLPS Propensity of m^6^A Regulators in PWNs

In addition to transcriptional regulation, several m^6^A regulators in PWN also displayed structural features associated with LLPS. LLPS propensity prediction revealed that two writers (BxRBM15a/b) and four readers (BxELAVL1a/b, BxFMR1, BxKHSRP) possess structural features associated with LLPS (Figure 6). BxRBM15a/b contain SPOC_SF domains, which have been demonstrated to participate in phase separation-mediated transcriptional regulation. In mammalian cells, the SPOC domain of SHARP cooperates with its intrinsically disordered regions (IDRs) to facilitate multivalent accumulation on Xist lncRNA, forming nuclear condensates [54]. Furthermore, BxELAVL1a/b, BxKHSRP, and BxFMR1 may drive phase separation through their intrinsically low-complexity domains [55]. In *D. melanogaster*, upon binding to m^6^A modified mRNAs, FMR1 undergoes LLPS, promoting the assembly and condensation of FMR1 ribonucleoprotein granules to regulate maternal mRNA decay during early embryogenesis [9]. Although PWN lacks YTH domain-containing readers, studies have shown that multivalent mRNAs containing multiple m^6^A sites can enhance phase separation by promoting interactions with RNA-binding proteins, thereby partitioning m^6^A modified mRNAs into membrane-less organelles such as P-bodies or stress granules, regulating mRNA stability and translation efficiency [36]. These findings provide a novel epitranscriptomic perspective for understanding the environmental adaptation mechanisms of PWNs. When PWN encounters environmental stresses such as low temperature, pine-derived metabolites, or host infection, m^6^A mediated phase separation may coordinate RNA condensate formation, thereby enabling rapid gene expression regulation for stress adaptation.

### 4.5. Limitations and Future Perspectives

Several limitations should be acknowledged. The expression data are transcriptomic and require protein-level validation. Functional roles of individual m^6^A regulators need further investigation using RNAi or CRISPR-based editing, and direct evidence of m^6^A modification on specific transcripts requires MeRIP-seq. Despite these limitations, this study provides the first systematic characterization of the m^6^A regulatory system in a plant–parasitic nematode, revealing non-canonical features that expand our understanding of epitranscriptomic regulation in parasitic nematodes. These non-canonical components may serve as novel targets for nematode-specific control strategies.

## 5. Conclusions

This study presents the genome-wide identification and systematic analysis of m^6^A regulators in PWNs. Through physicochemical property assessment, chromosomal localization, phylogenetic reconstruction, conserved motif and gene structure analysis, and expression profiling, 21 m^6^A regulators with evolutionarily conserved features were identified. These genes exhibited stage-specific expression patterns during the egg, larval, diapause, and adult stages, and showed significant responses to low temperature, β-pinene, and infection of *P. thunbergii* seedlings, indicating that m^6^A modification may function as an important regulatory mechanism integrating developmental and environmental signals in PWNs. Notably, the absence of canonical m^6^A erasers (FTO/ALKBH5) and YTH domain-containing readers, along with the selective retention of the ALKBH6/ALKBH8 subfamilies and KH/RRM domain-containing proteins, suggests that PWNs possess unique molecular mechanisms for dynamic m^6^A regulation, which establishes a crucial foundation for elucidating the functions of m^6^A modification in the development and stress adaptation of plant–parasitic nematodes. Future studies employing gene editing, MeRIP-seq, and other omics approaches will help elucidate the precise regulatory functions of key m^6^A regulators, thereby providing a theoretical basis for risk assessment of PWN dispersal and the development of control strategies.

## Figures and Tables

**Table 1:** Bioinformatic characterization of candidate m^6^A regulators in PWNs. This table summarizes the gene nomenclature, protein physicochemical properties (including protein size, molecular weight, pI, instability index, aliphatic index, GRAVY), and predicted subcellular localization.

Type	Gene Name	Gene ID	Protein Size (aa)	Molecular Weight (kDa)	Theoretical pI	Instability Index	Aliphatic Index	GRAVY	Subcellular Localization
Writers	*BxMETTL4*	BXYJ_LOCUS4230	687	76.89	6.27	49.23	94.51	−0.004	Plasma
	*BxMETTL5a*	BXYJ_LOCUS1798	225	25.72	4.88	39.26	90.09	−0.345	Cytoplasm
	*BxMETTL5b*	BXYJ_LOCUS2202	386	45.48	8.73	50.98	64.20	−0.897	Nucleus
	*BxMETTL14a*	BXYJ_LOCUS3394	345	40.03	5.70	44.85	76.64	−0.482	Nucleus
	*BxMETTL14b*	BXYJ_LCUS15221	284	32.39	5.87	34.83	93.73	−0.157	Cytoplasm
	*BxMETTL16*	BXYJ_LCUS15272	328	37.52	9.24	24.50	75.43	−0.454	Nucleus
	*BxZCCHC4*	BXYJ_LOCUS3322	437	50.91	9.35	28.30	72.04	−0.628	Cytoplasm
	*BxTMT1A*	BXYJ_LOCUS8277	219	24.97	9.07	25.72	77.99	−0.436	Cytoplasm
	*BxRBM15a*	BXYJ_LOCUS9155	509	58.28	9.26	48.56	66.44	−0.885	Nucleus
	*BxRBM15b*	BXYJ_LOCUS4315	2919	321.18	9.03	61.12	64.96	−0.807	Nucleus
Erasers	*BxALKBH6a*	BXYJ_LOCUS7963	259	29.03	8.94	48.23	88.42	−0.387	Nucleus
	*BxALKBH6b*	BXYJ_LOCUS4511	941	107.54	8.85	47.11	89.90	−0.461	Nucleus
	*BxALKBH8a*	BXYJ_LCUS10604	586	67.20	6.11	46.52	78.50	−0.426	Nucleus
	*BxALKBH8b*	BXYJ_LOCUS6797	548	62.96	7.67	46.86	80.44	−0.573	Extracellular
	*BxALKBH8c*	BXYJ_LOCUS7968	537	61.88	7.18	35.75	79.55	−0.566	Extracellular
	*BxALKBH8d*	BXYJ_LOCUS4761	748	86.04	5.40	32.40	83.68	−0.409	Extracellular
Readers	*BxELA* *V* *L1a*	BXYJ_LOCUS5335	471	50.13	8.84	44.25	83.21	−0.162	Nucleus
	*BxELA* *V* *L1b*	BXYJ_LOCUS6382	463	51.56	6.03	74.68	53.26	−0.884	Nucleus
	*BxFMR1*	BXYJ_LCUS13875	497	55.65	8.16	37.07	74.45	−0.740	Cytoplasm
	*BxKHSRP*	BXYJ_LCUS12119	522	55.67	8.57	44.90	70.84	−0.591	Cytoplasm
	*BxHNRNP*	BXYJ_LOCUS1542	364	40.00	9.17	47.45	75.30	−0.546	Cytoplasm

Note: All predictions are hypotheses requiring experimental validation.

## Data Availability

Data are contained within the article and Supplementary Materials.

## Supplementary Materials

The following supporting information can be downloaded at: https://www.mdpi.com/article/10.3390/biology15100786/s1, Table S1: Information on m^6^A homologues from PWNs and other species; Table S2: Primers used for qRT-PCR analysis in this study; Table S3: Secondary structure composition of m^6^A regulators in PWNs. The ratio indicates the proportion of amino acids forming each secondary structure relative to the total number of amino acids.

## Abbreviations

METTL3	Methyltransferase-like 3
METTL14	Methyltransferase-like 14
WTAP	Wilms tumor 1-associated protein
METTL16	Methyltransferase-like 16
FTO	Fat mass and obesity-associated protein
ALKBH5	AlkB homolog 5
HNRNPs	Heterogeneous nuclear ribonucleoproteins
IGF2BP1–3	Insulin-like growth factor 2 mRNA–binding proteins 1, 2, and 3
SAM	S-adenosylmethionine
pI	Isoelectric point
GRAVY	Grand average of hydropathicity
PrLDs	Prion-like domains
NJ	Neighbor-joining
